# Evaluation of the German biographic screening interview for fetal alcohol spectrum disorder (BSI-FASD)

**DOI:** 10.1038/s41598-021-83942-2

**Published:** 2021-03-04

**Authors:** Michael Widder, Luisa Mierzwa, Lina Schwerg, Henrike Schecke, Johannes Kornhuber, Polyxeni Bouna-Pyrrou, Jan Malte Bumb, Tanja Richter-Schmidinger, Bernd Lenz

**Affiliations:** 1 Department of Psychiatry and Psychotherapy, Friedrich-Alexander University Erlangen-Nürnberg (FAU), Schwabachanlage 6, 91054 Erlangen, Germany; 2 Sonnenhof e.V., Berlin, Germany; 3 Addiction Research Group, Department of Psychiatry and Psychotherapy, LVR-Klinikum Essen, Hospital of the University of Duisburg-Essen, Essen, Germany; 4 Department of Addictive Behavior and Addiction Medicine, Central Institute of Mental Health (CIMH), Medical Faculty Mannheim, Heidelberg University, Mannheim, Germany; 5 Feuerlein Center on Translational Addiction Medicine (FCTS), Heidelberg University, Mannheim, Germany

**Keywords:** Psychiatric disorders, Health care economics, Diagnosis, Preclinical research, Clinical trials, Neurological disorders, Disability

## Abstract

Alcohol consumption during pregnancy may lead to permanent damage in the offspring, including fetal alcohol spectrum disorders (FASD), which have an estimated prevalence of 1–8% worldwide. In adulthood, diagnosing FASD is time-consuming and costly. This study aimed to evaluate the discriminatory power of a German screening instrument for FASD in adults—the biographic screening interview (BSI-FASD). In an open-label comparative cohort study wherein a one-time survey was administered per participant, we compared 22 subjects with confirmed FASD with control groups of 15 subjects diagnosed with attention deficit hyperactivity disorder (ADHD), 20 subjects with alcohol or opiate dependence, 18 subjects with depression, and 31 controls without prenatal alcohol exposure. The BSI-FASD was found to be resource-efficient, user-friendly, comprehensible, and easily applicable. It provided an overall good convergent and discriminant validity with a sensitivity of 0.77 (adapted 0.86) and specificities between 0.70 and 1.00. The BSI-FASD subdomains differed in their power to differentiate FASD from the groups. This study established that the BSI-FASD is an efficient instrument to screen adults with suspected FASD. The BSI-FASD may facilitate future diagnostic evaluation and thereby contribute to improved treatment of affected individuals.

## Introduction

It has been well established for decades that alcohol consumption during pregnancy can cause permanent damage in the children’s development and behaviour^1^. Alcohol consumed during pregnancy can easily enter the fetus’ circulation via the placenta, causing cell damage and developmental disorders. In the literature, 1–8% of the population worldwide are affected by fetal alcohol spectrum disorders^2^, and 1.4–1.8% are affected in the European region^3^. Of those affected, facial malformations and growth abnormalities are found in 10–30%. FASD is also associated with heart defects, kidney damage, epilepsy, gastrointestinal diseases, and skeletal malformations^4^. Moreover, maternal alcohol consumption during pregnancy is believed to affect the offspring’s hormonal milieu, with effects on mental health lasting well into adulthood^5–7^.

FASD imposes a significant burden on the affected individual and on society at large. A national Swedish study found that FASD costs the healthcare system €76,000 annually per affected child (0–17 years) and €110,000 per affected adult (18–74 years)^8^. Those diagnosed with FASD suffer from a range of physiological, neurocognitive, and especially—behavioural impairments that negatively affect daily life functioning in various ways (e.g., academic achievement, adaptive social behaviour, independent living as an adult)^9^. Among other cognitive deficits, impairments in memory, learning, attention, and executive functions (e.g., self-inhibition) are found^10^. Further, there is an increased risk of school dropout, unemployment, substance abuse, unlawful behaviour, and comorbid psychiatric disorders^11^. Guidelines have already been developed for diagnosing FASD across the lifespan in various countries^10,11,12^. However, the available preventive, therapeutic, and supportive interventions remain extremely limited^13^.

Although a German S3 guideline for diagnosing FASD in children and adolescents has been published^14^, such guidelines for adults do not exist in German-speaking countries due to limited evidence. There is, as of yet, no generally accepted tool for adults to diagnose FASD, including fetal alcohol syndrome (FAS), partial FAS (pFAS), or alcohol-related neurodevelopmental disorder (ARND). Due to the lack of valid screening instruments, a plethora of different diagnostic approaches are used across medical centres. Moreover, only a small number of institutions offer appropriate diagnostics. Therefore, reliable screening instruments are strongly needed to accurately identify high-risk individuals for the expensive and time-consuming diagnostic procedures.

Subjects suffering from FASD present a diverse array of symptoms due to the different influences of prenatal alcohol exposure, social environment, education, support, and other possible factors. The aetiology of FASD is known and entirely preventable by alcohol abstinence during pregnancy, but in fact very difficult to diagnose. With regard to its relevance, there is surprisingly little research activity concerning FASD in adults. Studies addressing FASD are very likely to generate relevant health data.

Short palpebral fissure lengths, a smooth philtrum, and a thin upper lip are core diagnostic criteria for FAS and pFAS in children, all of which can be assessed with the FAS Facial Analysis Software developed by Astley and Clarren^15^. This tool offers an objective and resource-efficient approach to identify individuals with an FASD-typical facial morphology. However, (some) facial features are missing in ARND, so the applicability in these individuals is limited. Moreover, this tool may be less reliable for FAS diagnosis in adults because the characteristic facial and growth abnormalities often disappear with age^16^. The psychopathological overlap between FASD and other mental disorders (e.g., ADHD, affective disorders) further complicates diagnostic efforts.

The Life History Screen (LHS), which assesses nine domains (see below) that are often conspicuous among FASD patients, showed a sensitivity of 0.81 and a specificity of 0.66^17^. It was recently translated into German by Schwerg and colleagues, who developed the Biographical Screening Interview (BSI-FASD) after adapting it to the German context. They conducted a preliminary study comparing subjects with FASD to those suffering from alcohol dependence, which yielded quite promising results, with a sensitivity of 0.88 and a specificity of 0.94^18^. The standardized screening takes 5–8 min.

### Aims of the study

This study was designed to accomplish three goals. First, we aimed to obtain an initial impression of the instrument’s applicability. Second, we aimed to investigate the convergent and discriminant validity of the BSI-FASD using groups with FASD, attention deficit hyperactivity disorder (ADHD), alcohol or opiate dependence, depression, and controls whose mothers did not consume alcohol during pregnancy. Third, we aimed to compare the results of the BSI-FASD and those of the FAS Facial Analysis Software to discriminate subjects with FASD from those without FASD.

## Materials and methods

### Study design and recruitment setting

We conducted an open-label comparative cohort study with a one-time recruitment (~ 30 min) per participant (Trial Registration: German Clinical Trials Register identifier DRKS00017174). The subjects were enrolled from April 2019 to December 2019 at the Department of Psychiatry and Psychotherapy of the Friedrich-Alexander University Erlangen-Nürnberg (FAU). Participants met the inclusion criteria if they were between 18 and 64 years of age. Informed consent was obtained from all subjects. General exclusion criteria for all groups were pregnancy, lactation period, or the refusal of written informed consent. This study was approved by the Ethics Committee of the Medical Faculty of the Friedrich-Alexander University Erlangen-Nürnberg (116_19 B). All methods were performed in accordance with relevant guidelines and regulations.

### Screening and recruitment procedures

The study population included subjects recruited from the following groups:

(1) 22 subjects with confirmed FASD (FAS *n* = 5, pFAS *n* = 10, ARND *n* = 7) from a specialized outpatient FASD setting, who each attended approximately six 90-min appointments. In the medical examination, a detailed psychiatric anamnesis was obtained, including everyday problems, neurological symptoms, the subjects’ psychosocial history, and alcohol consumption behaviour of their parents. Childhood pictures, the present facial physiognomy, and body measurements were analysed concerning diagnostic criteria. With neuropsychological instruments, the cognitive domains of intelligence, memory, attention, and executive functions were tested. In addition, we examined whether personality disorders, ADHD, PTSD, or depression were present. Following diagnosis, we held interdisciplinary conferences to discuss the findings; the results were documented in a detailed medical report.

The diagnosis was confirmed based on the German, Canadian, and Australian guidelines. The following criteria were used: the presence of growth disorders, sentinel facial features, central nervous system (CNS) abnormalities, and prenatal alcohol exposure. For the diagnosis of FAS, the criteria of growth disorders (birth weight or body weight < 10th percentile, birth length or body length < 10th percentile, or BMI < 10th percentile), all three sentinel facial features (i.e., short palpebral fissures, smooth or flattened philtrum, thin upper lip), and CNS abnormalities (significant abnormalities in at least three neurodevelopmental domains) had to be met. The presence of alcohol consumption during pregnancy was optional for the diagnosis of FAS. To diagnose pFAS, at least two sentinel facial features, CNS abnormalities, and at least a likely consumption of alcohol during pregnancy had to be present. For diagnosis of ARND, the criteria of CNS abnormalities and confirmed alcohol consumption during pregnancy had to be met. Additional data, which were recommended in the guidelines, were considered.

(2) 15 patients with confirmed ADHD (ICD-10: F90.0) from our outpatient ADHD setting. The diagnosis was made according to ICD-10 with the following well-validated tests: the short form of the Wender Utah Rating Scale^19^, ADHD self-questionnaire “ADHS-SB”^20^, and the Wender-Reimherr Interview^21^. Supplementary data were gathered from anamnesis by caregivers and from primary school certificates. To determine deviating personality dimensions, the “Inventar Klinischer Persönlichkeitsakzentuierungen”^22^ was used and for depressive symptoms the Beck Depression Inventory-II^23^.

(3) 11 patients with the primary diagnosis of alcohol dependence (ICD-10: F10.2) from psychiatric wards, who were admitted for withdrawal treatment, and nine outpatients with opiate dependence (ICD-10: F11.2) from the department’s Opioid Maintenance Therapy program (*n* = 20).

(4) 18 patients with a moderate-to-severe depressive disorder (ICD-10: F33.2, *n* = 13; F32.2, *n* = 2; F33.1, *n* = 3) from psychiatric wards. After discharge, three patients were diagnosed with a less severe depression, which we did not exclude because of their overall severity level due to comorbidities. Four patients did not meet the criteria for a depressive disorder after discharge and were, therefore, excluded.

(5) 31 control subjects recruited by accessing mothers of adult offspring. The mothers reported having been abstinent from alcohol during pregnancy (or a total abstinence from alcohol across the lifetime). The control subjects without prenatal alcohol exposure were recruited via flyers and social media or asked by their children themselves.

### The BSI-FASD

The German BSI-FASD version was initially developed by Schwerg and colleagues on the basis of the English LHS^17^. The development of the BSI-FASD was a five step process. First, Schwerg translated the LHS into German. Second, the German translation was sent to the LHS authors for retranslation, thus ensuring that no meaning or information was lost or compromised during the translation process. During the third step, some items were adjusted in order to adopt them to the German culture. For example, the LHS item that asks respondents for their highest school year completed results in a red flag if the response is “grade 10 or less”, since this indicates that the person left school without a diploma. In Germany however, completing school through the 10th grade is quite a good qualification and not a red flag in any way. Thus, the item needed to be adjusted to have the red flag response represent the initial intent: unfinished schooling. In Germany, an appropriate application of the red flag response would occur if the respondent completed 8 years or less of school. In the fourth step, Schwerg and an FASD-expert, who suffers from FASD herself, checked each item for comprehensibility. For some items, the FASD-expert suggested paraphrases, which were added to the Screening as B-Notes. In the fifth step, the German-translated and -adapted BSI-FASD was evaluated by administering it to N = 111 subjects with promising results in specificity (0.94) and sensitivity (0.88)^18,24^. The BSI-FASD is a structured interview with 30 scored items divided into nine domains: Childhood History* (2 items), Maternal Alcohol Use* (3 items), Education (4 items), Criminal History (3 items), Substance Use (2 items), Employment and Income (2 items), Living Situation (2 items), Mental Health (3 items), and Day-to-Day Behaviour* (11 items). Each domain can be red-flagged by a predetermined number of red-flag responses. Subjects who received red flags in all three “Key Life History Domains” (*), or in two such domains and two or more other domains, are classified as positive and should be referred for further FASD diagnostics. Importantly, the interviewers (C.R. and L.M.) complied rigorously with the exact wording of the screening interview. If the subjects did not understand a question, we used the “B-note” (a predetermined paraphrase) for each question. Overall, previous studies reported that the interview is an easy and quick diagnostic tool^17,24^. The interviews were conducted by well-trained physicians and doctoral students.

### FAS facial analysis software

The pictures required for software analysis were taken and evaluated by well-trained physicians and doctoral students. Photographs of the subjects’ faces were taken in frontal view, ¾ view, and lateral view. Afterwards, the pictures were analysed for palpebral fissure length, smooth philtrum, and thin upper lip by the FAS Facial Analysis Software, which was developed by Hemingway (University of Washington) in 2003 and which was later used in diagnostics within the 4-Digit Code for FASD by Astley and Clarren^15^.

### Statistical analyses

Data were analysed with SPSS for Windows 24.0 (SPSS Inc., Chicago, IL). Group comparisons were performed using the Mann–Whitney U test. We applied χ^2^ tests for differences in the frequencies and report *p *values from two-tailed Fisher’s exact test if at least one cell failed to reach an expected value of five observations. Binary logistic regression analysis (FSTEP(LR)) was employed to evaluate combinations of different BSI-FASD domains. We used receiver operating characteristic (ROC) curve analysis to estimate the cut-point values of BSI-FASD domains to separate subjects from the FASD group from those from the other groups (Youden cut-point, sensitivity, and specificity). Values of *p* < 0.05 for two-tailed tests were considered significant.

### Ethical approval

This study was approved by the Ethics Committee of the Medical Faculty of the Friedrich-Alexander University Erlangen-Nürnberg (116_19 B).

## Results

### Sociodemographic characteristics

The FASD group did not significantly differ from the control groups (ADHD, alcohol/opioid dependence, depression, controls without prenatal alcohol exposure) with respect to sex or age, except for fewer females in the ADHD group and older age in the alcohol/opioid dependencies groups (Table 1). Sex or age were not significantly related to the number of positive BSI-FASD Key Life History Domains, positive BSI-FASD Other Domains, or the FAS Facial Analysis Software score, except for a negative correlation between age and positive BSI-FASD Other Domains in the group of alcohol/opioid-dependent subjects (Table S1).

**Table 1:** Sociodemographic characteristics, positive BSI-FASD domains, and FAS Facial Analysis Software results in participants with FASD, ADHD, alcohol/opioid dependencies (AOD), depression (DEP), and without prenatal alcohol exposure (WPAE).

		FASD	Controls			
			ADHD	AOD	DEP	WPAE
N		22	15	20	18	31
**Sex**	Males (n)	11	13	12	6	13
	Females (n)	11	2	8	12	18
Versus FASD	χ^2^		5.3	0.4	1.1	0.3
	p^a^		**0.022**	0.516	0.289	0.561
**Age (years)**	Mean	27	29	39	25	24
	SD	6	4	9	5	2
	Median	26	30	39	26	24
	IQR	21	27	32	22	23
		32	32	48	28	26
Versus FASD	U		123	70	173	290
	p^b^		0.193	**< 0.001**	0.487	0.355
**BSI-FASD screening result**	Positive (n)	17	1	6	2	0
	Negative (n)	5	14	14	16	31
**Sensitivity**		0.77				
**Specificity**			0.93	0.70	0.89	1.00
Versus FASD	χ^2^		17.8	9.5	17.4	35.3
	p^a^		**< 0.001**	**0.002**	** < 0.001**	** < 0.001**
**Positive BSI-FASD key life History domains**	0 (n)	0	4	9	7	30
	1 (n)	5	9	5	9	1
	2 (n)	12	2	6	1	0
	3 (n)	5	0	0	1	0
Versus FASD	U		45	79	53	3
	p^b^		** < 0.001**	**< 0.001**	**< 0.001**	**< 0.001**
**Positive BSI-FASD other domains**	0 (n)	0	1	1	2	11
	1 (n)	0	5	0	3	14
	2 (n)	3	4	5	8	3
	3 (n)	9	2	9	4	3
	4 (n)	6	2	1	1	0
	5 (n)	3	1	4	0	0
	6 (n)	1	0	0	0	0
Versus FASD	U		71	168	57	27
	p^b^		**0.003**	0.169	**< 0.001**	**< 0.001**
**FAS facial analysis software score**	At least mild (n)	12	5	10	3	9
	Absent (n)	6	8	8	11	21
**Sensitivity**		0.67				
**Specificity**			0.62	0.44	0.79	0.70
Versus FASD	χ^2^		2.4	0.5	6.5	6.1
	p^a^		0.119	0.494	**0.011**	**0.013**
**FAS facial analysis software**	Absent (n)	6	8	8	11	21
	Mild (n)	8	5	8	3	9
	Moderate (n)	2	0	2	0	0
	Severe (n)	2	0	0	0	0
Versus FASD	U		74	134	63	153
	p^b^		0.060	0.337	**0.008**	**0.005**

The table shows absolute frequencies and mean, standard deviation (SD), median, and interquartile range (IQR). *P* values are derived from ^a^χ^2^ and ^b^Mann–Whitney U tests. *P* < 0.05 in bold.

### BSI-FASD and FAS facial analysis software results

Overall, the BSI-FASD and the FAS Facial Analysis Software proved to be resource-efficient, user-friendly, comprehensible, and easily applicable. The interview averaged 5–8 min in duration. The FASD group was significantly more likely to be screened as positive by the BSI-FASD than were the ADHD subjects, the alcohol/opioid-dependent subjects, the depressed subjects, and the participants without prenatal alcohol exposure. The BSI-FASD showed a sensitivity of 0.77 and specificities ranging from 0.70 for the alcohol/opioid-dependent group to 1.00 for the group without prenatal alcohol exposure. Moreover, the FASD group showed significantly more positive Key Life History Domains and Other Domains than the control groups, except for the Other Domains in the alcohol/opioid-dependent group.

The FAS Facial Analysis Software revealed at least one facial sign of FAS in the FASD group significantly more often than in the subjects with depression and the group without prenatal alcohol exposure. We found a sensitivity of 0.67 and specificities of 0.79 for the depression group and 0.70 for the group without prenatal alcohol exposure. The FAS Facial Analysis Software score did not significantly differ between the FASD group and either the ADHD group or the alcohol/opioid-dependent group (see Table 1 for further details).

### BSI-FASD subdomains

In subsequent BSI-FASD subdomain analyses, only Maternal Alcohol Use* consistently and significantly differentiated the FASD group from the control groups. The other two Key Life History Domains, namely, Childhood History* and Day-to-Day-Behaviour*, were able to significantly differentiate the FASD group from the dependence group and the control group without prenatal alcohol exposure, but not from the ADHD group and the depression group. From the remaining six other domains, Education and Living Situation were able to significantly differentiate the FASD group from three of the four control groups particularly well. The domain of Criminal History significantly differentiated the FASD group from two of the four control groups, the domains of Mental Health and of Employment and Income from one of the four control groups. The domain of Substance Use did not significantly differ between the FASD group and any control group.

The sensitivities for red-flagged domains ranged from 0.18 for Substance Use to 0.95 for Maternal Alcohol Use*, and the specificities ranged from 0.15 to 0.74 for Employment and Income to 0.89 to 1.00 for Childhood History* (see Table 2 for further details).

**Table 2:** BSI-FASD Key Life History Domains (*) and Other Domains in participants with FASD, ADHD, alcohol/opioid dependencies (AOD), depression (DEP), and without prenatal alcohol exposure (WPAE).

	Red flag	FASD	Controls			
			ADHD	AOD	DEP	WPAE
N		22	15	20	18	31
Childhood history*	Yes (n)	6	0	0	2	0
	No (n)	16	15	20	16	31
Sensitivity		0.27				
Specificity			1.00	1.00	0.89	1.00
Versus FASD	χ^2^		4.9	6.4	1.6	9.5
	p^a^		0.063	**0.022**	0.258	**0.003**
Maternal alcohol use*	Yes (n)	21	3	9	3	0
	No (n)	1	12	11	15	31
Sensitivity		0.95				
Specificity			0.80	0.55	0.83	1.00
Versus FASD	χ^2^		22.3	13.1	25.6	49.0
	p^a^		**< 0.001**	**< 0.001**	**< 0.001**	**< 0.001**
Day-to-day behaviour*	Yes (n)	17	10	8	9	1
	No (n)	5	5	12	9	30
Sensitivity		0.77				
Specificity			0.33	0.60	0.50	0.97
Versus FASD	χ^2^		0.5	6.0	3.2	31.5
	p^a^		0.708	**0.014**	0.072	**< 0.001**
**Education**	Yes (n)	19	7	15	5	10
	No (n)	3	8	5	13	21
Sensitivity		0.86				
Specificity			0.53	0.25	0.72	0.68
Versus FASD	χ^2^		6.7	0.9	14.2	15.2
	p^a^		**0.025**	0.445	**< 0.001**	**< 0.001**
**Criminal history**	Yes (n)	17	8	16	3	2
	No (n)	5	7	4	15	29
Sensitivity		0.77				
Specificity			0.47	0.20	0.83	0.94
Versus FASD	χ^2^		2.3	< 0.1	14.5	28.1
	p^a^		0.164	1.000	**< 0.001**	**< 0.001**
**Substance use**	Yes (n)	4	2	5	1	2
	No (n)	18	13	15	17	29
Sensitivity		0.18				
Specificity			0.87	0.75	0.94	0.94
Versus FASD	χ^2^		0.2	0.3	1.4	1.8
	p^a^		1.000	0.714	0.355	0.219
**Employment and income**	Yes (n)	15	9	17	10	8
	No (n)	7	6	3	8	23
Sensitivity		0.68				
Specificity			0.40	0.15	0.44	0.74
Versus FASD	χ^2^		0.3	1.6	0.7	9.4
	p^a^		0.609	0.284	0.412	**0.002**
**Living situation**	Yes (n)	12	1	1	7	7
	No (n)	10	14	19	11	24
Sensitivity		0.55				
Specificity			0.93	0.95	0.61	0.77
Versus FASD	χ^2^		9.0	12.0	1.0	5.7
	p^a^		**0.003**	**0.001**	0.324	**0.017**
**Mental health**	Yes (n)	11	5	7	9	0
	No (n)	11	10	13	9	31
Sensitivity		0.50				
Specificity			0.67	0.65	0.50	1.00
Versus FASD	χ^2^		1.0	1.0	0.0	19.6
	p^a^		0.315	0.327	1.000	**< 0.001**

The table shows absolute frequencies. *P* values are derived from ^a^χ^2^ or Fisher tests. *P* < 0.05 in bold.

We conducted stepwise binary logistic regression analysis to evaluate different combinations of BSI-FASD domains. The following predictors were included in the model following the last step: (1) Maternal Alcohol Use*, (2) Education, and (3) Living Situation in the FASD vs. ADHD model, (1) Childhood History*, (2) Maternal Alcohol Use*, and (3) Living Situation in the FASD vs. alcohol/opioid-dependent patients model, (1) Maternal Alcohol Use*, (2) Criminal History, (3) Living Situation in the FASD vs. depressed patients model, and (1) Maternal Alcohol Use* and (2) Day-to-Day Behaviour* in the FASD vs. subjects without prenatal alcohol exposure model. This shows that the method to recommend further diagnostic procedures in case of red flags in at least two Key Life History Domains, as suggested by Grant et al*.* and Schwerg et al*.*, may be feasible to differentiate FASD from alcohol/opioid-dependent patients and controls without prenatal alcohol exposure, but less reliable for patients with ADHD or depression^17,24^.

For Maternal Alcohol Use*, Day-to-Day Behaviour*, Education, and all Other Domains, ROC curve analysis using Youden indexes confirmed cut-off points described by Grant et al*.* and Schwerg^17,24^ (Table S2). For discrimination from the cohorts with mental disorders, the Youden cut-point was sometimes slightly higher in terms of Maternal Alcohol Use, Day-to-Day Behaviour, Education, Employment and Income, and Living Situation. Interestingly, the Youden cut-point was lower for the Childhood History domain (≥ 1.5) for all groups than what has been previously described^17,24^. Thus, we adapted the classification method using the ≥ 2 cut-off point, re-analysed the data, and found an increased and good sensitivity of 0.86 and specificities between 0.70 for the alcohol/opioid-dependent group and 1.00 for the group without prenatal alcohol exposure (see Table 3).

**Table 3:** Re-analysis using the adapted cut-off point for the Childhood History Domain.

		FASD	Controls			
			ADHD	AOD	DEP	WPAE
N		22	15	20	18	31
**BSI-FASD screening result**	Positive (n)	19	2	6	2	0
	Negative (n)	3	13	14	16	31
**Sensitivity**		0.86				
**Specificity**			0.87	0.70	0.89	1.00
Versus FASD	χ^2^		19.4	13.8	22.5	41.7
	p^a^		**< 0.001**	**< 0.001**	**< 0.001**	**< 0.001**

The table shows absolute frequencies. *P* values are derived from ^a^χ^2^ tests. *P* < 0.05 in bold.

## Discussion

Due to the lack of an established German screening instrument, diagnosing FASD in adults is significantly time-consuming. Specialized centres conduct multiple sessions with different medical and neuropsychological tests that need many hours. In our study, the BSI-FASD was found to be a user-friendly, comprehensible, and resource-efficient instrument to screen for FASD. One BSI-FASD interview averaged 5–8 min in duration.

To our knowledge, this investigation is the first to assess the psychometric properties of the German BSI-FASD in samples with various mental disorders and in control subjects without prenatal alcohol exposure. Notably, the BSI-FASD did not result in any false positive in the group of control subjects without prenatal alcohol exposure. This highlights that a positive BSI-FASD is strongly related to mental disorders. We also provide evidence that subdomains differ in their power to differentiate FASD from ADHD, dependence, depression, and controls without prenatal alcohol exposure. Our study confirmed the findings from Schwerg^24^ that most of the domains of the BSI-FASD have medium-to-high differential value. We also confirmed that the domain Employment and Income seems to be of very limited differential value, and that the domain Substance Use appears to yield no differential value. However, the domain Criminal History previously identified as not being able to differentiate between FASD and non-FASD subjects^24^, proved to be of significant differential value in two of our four control groups.

We found good BSI-FASD specificities (> 0.80) for all control groups, with the exception of the alcohol/opioid-dependent group (0.70). This lower specificity may be due to a number of alcohol/opioid-dependent patients with comorbid FASD in our sample as we did not rule out prenatal alcohol exposure in the clinical control groups. Given that alcohol dependence is highly heritable, it is not unlikely that mothers of alcohol-dependent patients themselves suffered from an alcohol use disorder and, thus, drank alcohol during pregnancy. Also, substance use disorders are well known to trail a stronger downward drift in socioeconomic status relative to affective disorders and personality disorders^9^. Thus, the lower specificity of the BSI-FASD in the alcohol/opioid dependence group might be imparted by socioeconomic status, which is also known to be lower for people affected by FASD^25^. The analysis suggests that the domains (1) Childhood History*, (2) Maternal Alcohol Use*, and (3) Living Situation may be particularly relevant for the dependence group. The high specificity of 0.93 (0.87 for the version adapted to the decreased cut-off for the Childhood History* domain because of the improved sensitivity) in the ADHD group is particularly notable since attention deficit and hyperactivity symptoms are also often found in subjects with FASD^26^. Our results suggest that the BSI-FASD may complement the clinical differential diagnosis between FASD and ADHD, and thus optimize the diagnostic accuracy of ADHD patients. In particular, it may lead to an individualized therapy for subjects with ADHD, ADHD/FASD, and FASD.

We found a sensitivity of 0.77 using the classification method previously described by the teams of Grant and Schwerg^17,18^. After ROC curve analysis, we increased the cut-point of the Key Life History Domain of Childhood History to ≥ 2, re-analysed the data, and found an increased sensitivity of 0.86. Thus, we recommend the ≥ 2 threshold in future studies administering the German BSI-FASD. Overall, we found that the sensitivity of the BSI-FASD is comparable to that of the original English LHS^17^ and the previous German version^24^.

It is well established that the facial abnormalities characteristic of FASD decrease with older age. The BSI-FASD was superior to the FAS Facial Analysis Software in both sensitivity and specificity. This agrees with the observation that the typical facial and growth abnormalities relevant to diagnose FASD in under-aged individuals become less prominent with older age. However, combinations of BSI-FASD and FAS Facial Analysis Software domains might be even more sensitive and specific than the BSI-FASD alone. Due to the small sample size of this project, we were not able to further explore this idea. Future studies addressing this issue are needed.

Our results are not only relevant to the diagnostic optimization of subjects with FASD, but also to their medical care. With its efficiency and reliability, the BSI-FASD will help to correctly allocate patients with FASD to targeted treatments such as cognitive rehabilitation^27,28^.

We did not further investigate whether those from the ADHD, alcohol/opioid dependencies, and depression groups, who were classified as positive by the BSI-FASD, were also true positives (i.e., whether they also fulfilled the FASD criteria), or were exposed to alcohol prenatally. This may have even resulted in an underestimation of the true specificity rates, and the issue should be addressed in further studies. The findings of this study may not generalize to individuals diagnosed with FASD using other criteria. In addition, the relatively small sample sizes certainly limit the generalizability of our results. The raters were not blinded to group allocations, which may have introduced bias, and should also be considered in future studies. However, we complied rigorously with the exact wording of the structured BSI-FASD. Moreover, other psychiatric comorbidities (e.g., personality disorders) and ethnicity may have influenced the results in separate domains.

We wish to highlight that we were particularly cautious to assure that the mothers of participants from the control group without prenatal alcohol exposure did not drink alcohol during pregnancy. Moreover, we made great efforts and conducted comprehensive diagnostic procedures to diagnose FASD, ADHD, depression, and alcohol/opioid dependencies for a reliable demarcation between FASD and other psychiatric disorders, which may have overlapping symptoms. The groups were well-matched regarding sex and age, with minor exceptions.

In summary, our data support that the German BSI-FASD is a resource-efficient, user-friendly, comprehensible, and easily applicable screening instrument with high sensitivity and specificity. This study demonstrates that the BSI-FASD is an efficient tool to select subjects that should be referred to the limited number of specialized centres for adult FASD diagnosis. Moreover, it should be used more broadly to screen for FASD in German-speaking risk cohorts with ADHD, affective disorders, and alcohol/opioid dependencies to enable earlier and more individualized treatments. The BSI-FASD builds a basis for new studies regarding diagnostics and therapeutic aims.

## Supplementary information

Supplementary tables.
