# Supplementary material for: Evaluation of the German biographic screening interview for fetal alcohol spectrum disorder (BSI-FASD)
Source: Sci Rep. 2021 Mar 4;11:5233. doi: 10.1038/s41598-021-83942-2 (PMC7933170; doi:10.1038/s41598-021-83942-2)
Supplement: Supplementary file 1 — Supplementary tables. [file 41598_2021_83942_MOESM1_ESM.docx]

Supplementary material

**Evaluation of the German Biographic Screening Interview for Fetal Alcohol Spectrum Disorder (BSI-FASD)**

Running title: BSI-FASD

Michael Widder*^,1^, MD, Luisa Mierzwa^1^, Cand. med., Lina Schwerg^2^, M.Sc., Henrike Schecke^3^, PhD, Johannes Kornhuber^1^ (ORCID 0000-0002-8096-3987), MD, Polyxeni Bouna-Pyrrou^1^, MD, Malte Bumb^4,5^, MD, Tanja Richter-Schmidinger^1^ (0000-0001-5426-8342), PhD, Bernd Lenz^1,4,5^ (ORCID 0000-0001-6086-0924), MD

^1^Department of Psychiatry and Psychotherapy, Friedrich-Alexander University Erlangen-Nürnberg (FAU), Germany. ^2^Sonnenhof e.V., Berlin, Germany. ^3^Addiction Research Group at the Department of Psychiatry and Psychotherapy, LVR-Klinikum Essen, Hospital of the University of Duisburg-Essen, Essen, Germany. ^4^Department of Addictive Behavior and Addiction Medicine, Central Institute of Mental Health (CIMH), Medical Faculty Mannheim, Heidelberg University, Germany. ^5^Feuerlein Center on Translational Addiction Medicine (FCTS), Heidelberg University, Germany.

*Corresponding author: Department of Psychiatry and Psychotherapy, Friedrich-Alexander University Erlangen-Nürnberg (FAU), Schwabachanlage 6, 91054 Erlangen, Germany

Phone: +49 9131 85-33001, Fax: +49 9131 85-34105

E-mail: [michael.widder@uk-erlangen.de](mailto:michael.widder@uk-erlangen.de)

| Table S1. Associations of sex and age with BSI-FASD Key Life History Domains, BSI-FASD Other Domains, and FAS Facial Analysis Software score | | | | | | | | | | | | | | | |
| --- | --- | --- | --- | --- | --- | --- | --- | --- | --- | --- | --- | --- | --- | --- | --- |
| Original | Positive BSI-FASD Key Life History Domains | | | | | Positive BSI-FASD Other Domains | | | | | FAS Facial Analysis Software score | | | | |
|  | FASD | ADHD | AOD | Depression | WPAE | FASD | ADHD | AOD | Depression | WPAE | FASD | ADHD | AOD | Depression | WPAE |
| N | 22 | 15 | 20 | 18 | 31 | 22 | 15 | 20 | 18 | 31 | 18 | 13 | 18 | 14 | 30 |
| Sex |  |  |  |  |  |  |  |  |  |  |  |  |  |  |  |
| U | 35 | 9 | 43 | 27 | 108 | 40 | 12 | 46 | 34 | 110 | 39 | 3 | 27 | 9 | 87 |
| p | 0.064 | 0.383 | 0.679 | 0.351 | 0.239 | 0.158 | 0.861 | 0.870 | 0.843 | 0.763 | 0.924 | 0.062 | 0.204 | 0.442 | 0.263 |
| Age |  |  |  |  |  |  |  |  |  |  |  |  |  |  |  |
| ρ | -0.160 | 0.311 | -0.364 | -0.135 | 0.145 | -0.202 | 0.141 | -0.620 | -0.273 | -0.318 | 0.062 | -0.064 | 0.460 | -0.196 | -0.247 |
| p | 0.478 | 0.259 | 0.115 | 0.594 | 0.437 | 0.368 | 0.616 | **0.004** | 0.272 | 0.081 | 0.807 | 0.837 | 0.054 | 0.501 | 0.188 |
| This table shows ^a^Mann-Whitney U tests and ^b^Spearman correlations. FASD, ADHD, alcohol/opioid dependencies (AOD), depression (DEP), and without prenatal alcohol exposure (WPAE). *P* < 0.05 in bold. | | | | | | | | | | | | | | | |

| Table S2. | | | | | | | | | | | | | | | | | | | | |
| --- | --- | --- | --- | --- | --- | --- | --- | --- | --- | --- | --- | --- | --- | --- | --- | --- | --- | --- | --- | --- |
|  | Combined | | | | ADHD | | | | AOD | | | | Depression | | | | WPAE | | | |
|  | Cut-point | Sens | Spec | Youden index | Cut-point | Sens | Spec | Youden index | Cut-point | Sens | Spec | Youden index | Cut-point | Sens | Spec | Youden index | Cut-point | Sens | Spec | Youden index |
| Childhood History* | 0.0 | 1.00 | 0.00 | 1.00 | 0.0 | 1.00 | 0.00 | 1.00 | 0.0 | 1.00 | 0.00 | 1.00 | 0.0 | 1.00 | 0.00 | 1.00 | 0.0 | 1.00 | 0.00 | 1.00 |
| *Schwerg et al.: >2* | 1.5 | 0.50 | 0.88 | 1.38^#^ | 1.5 | 0.50 | 0.80 | 1.30^#^ | 1.5 | 0.50 | 0.90 | 1.40^#^ | 1.5 | 0.50 | 0.78 | 1.28^#^ | 1.5 | 0.50 | 0.97 | 1.47^#^ |
|  | 2.5 | 0.27 | 0.98 | 1.25 | 2.5 | 0.27 | 1.00 | 1.27 | 2.5 | 0.27 | 1.00 | 1.27 | 2.5 | 0.27 | 0.89 | 1.16 | 2.5 | 0.27 | 1.00 | 1.27 |
|  | 3.5 | 0.18 | 0.99 | 1.17 | 3.5 | 0.18 | 1.00 | 1.18 | 3.5 | 0.18 | 1.00 | 1.18 | 3.5 | 0.18 | 0.94 | 1.13 | 3.5 | 0.18 | 1.00 | 1.18 |
|  | 4.5 | 0.05 | 0.99 | 1.03 | 4.5 | 0.05 | 1.00 | 1.05 | 4.5 | 0.05 | 1.00 | 1.05 | 4.5 | 0.05 | 0.94 | 0.99 | 4.5 | 0.05 | 1.00 | 1.05 |
|  | 5.5 | 0.00 | 0.99 | 0.99 | 6.0 | 0.00 | 1.00 | 1.00 | 6.0 | 0.00 | 1.00 | 1.00 | 5.5 | 0.00 | 0.94 | 0.94 | 6.0 | 0.00 | 1.00 | 1.00 |
|  | 7.0 | 0.00 | 1.00 | 1.00 |  |  |  |  |  |  |  |  | 7.0 | 0.00 | 1.00 | 1.00 |  |  |  |  |
| Maternal Alcohol Use* | -1.0 | 1.00 | 0.00 | 1.00 | -1.0 | 1.00 | 0.00 | 1.00 | -1.0 | 1.00 | 0.00 | 1.00 | -1.0 | 1.00 | 0.00 | 1.00 | -1.0 | 1.00 | 0.00 | 1.00 |
|  | 0.5 | 1.00 | 0.54 | 1.54 | 0.5 | 1.00 | 0.53 | 1.53 | 0.5 | 1.00 | 0.40 | 1.40 | 0.5 | 1.00 | 0.56 | 1.56 | 0.5 | 1.00 | 0.61 | 1.61 |
| *Schwerg et al.: ≥2* | 1.5 | 0.95 | 0.82 | 1.78 | 1.5 | 0.95 | 0.80 | 1.75 | 1.5 | 0.95 | 0.55 | 1.50 | 1.5 | 0.95 | 0.83 | 1.79 | 1.5 | 0.95 | 1.00 | 1.95^#^ |
|  | 2.5 | 0.91 | 0.90 | 1.81^#^ | 2.5 | 0.91 | 0.87 | 1.78^#^ | 2.5 | 0.91 | 0.75 | 1.66^#^ | 2.5 | 0.91 | 0.94 | 1.85^#^ | 2.5 | 0.91 | 1.00 | 1.91 |
|  | 4.0 | 0.00 | 1.00 | 1.00 | 4.0 | 0.00 | 1.00 | 1.00 | 4.0 | 0.00 | 1.00 | 1.00 | 4.0 | 0.00 | 1.00 | 1.00 | 4.0 | 0.00 | 1.00 | 1.00 |
| Day-to-Day Behaviour* | -1.0 | 1.00 | 0.00 | 1.00 | 0.0 | 1.00 | 0.00 | 1.00 | 0.0 | 1.00 | 0.00 | 1.00 | 0.0 | 1.00 | 0.00 | 1.00 | -1.0 | 1.00 | 0.00 | 1.00 |
|  | 0.5 | 1.00 | 0.13 | 1.13 |  |  |  |  |  |  |  |  |  |  |  |  | 0.5 | 1.00 | 0.35 | 1.35 |
|  | 1.5 | 1.00 | 0.33 | 1.33 | 1.5 | 1.00 | 0.07 | 1.07 | 1.5 | 1.00 | 0.15 | 1.15 | 1.5 | 1.00 | 0.11 | 1.11 | 1.5 | 1.00 | 0.71 | 1.71 |
|  | 2.5 | 0.86 | 0.44 | 1.30 | 2.5 | 0.86 | 0.07 | 0.93 | 2.5 | 0.86 | 0.30 | 1.16 | 2.5 | 0.86 | 0.22 | 1.09 | 2.5 | 0.86 | 0.84 | 1.70 |
|  | 3.5 | 0.77 | 0.56 | 1.33 | 3.5 | 0.77 | 0.20 | 0.97 | 3.5 | 0.77 | 0.40 | 1.17 | 3.5 | 0.77 | 0.39 | 1.16 | 3.5 | 0.77 | 0.94 | 1.71 |
| *Schwerg et al.: ≥5* | 4.5 | 0.77 | 0.67 | 1.44^#^ | 4.5 | 0.77 | 0.33 | 1.11 | 4.5 | 0.77 | 0.60 | 1.37 | 4.5 | 0.77 | 0.50 | 1.27 | 4.5 | 0.77 | 0.97 | 1.74^#^ |
|  | 5.5 | 0.59 | 0.75 | 1.34 | 5.5 | 0.59 | 0.53 | 1.12 | 5.5 | 0.59 | 0.65 | 1.24 | 5.5 | 0.59 | 0.61 | 1.20 | 5.5 | 0.59 | 1.00 | 1.59 |
|  | 6.5 | 0.50 | 0.89 | 1.39 | 6.5 | 0.50 | 0.93 | 1.43^#^ | 6.5 | 0.50 | 0.75 | 1.25^#^ | 6.5 | 0.50 | 0.83 | 1.33^#^ | 6.5 | 0.50 | 1.00 | 1.50 |
|  | 7.5 | 0.36 | 0.95 | 1.32 | 7.5 | 0.36 | 1.00 | 1.36 | 7.5 | 0.36 | 0.85 | 1.21 | 7.5 | 0.36 | 0.94 | 1.31 | 7.5 | 0.36 | 1.00 | 1.36 |
|  | 8.5 | 0.23 | 0.98 | 1.20 | 8.5 | 0.23 | 1.00 | 1.23 | 8.5 | 0.23 | 0.90 | 1.13 | 8.5 | 0.23 | 1.00 | 1.23 | 8.5 | 0.23 | 1.00 | 1.23 |
|  | 9.5 | 0.09 | 0.98 | 1.07 | 9.5 | 0.09 | 1.00 | 1.09 | 9.5 | 0.09 | 0.90 | 0.99 | 9.5 | 0.09 | 1.00 | 1.09 | 9.5 | 0.09 | 1.00 | 1.09 |
|  | 11.0 | 0.00 | 1.00 | 1.00 | 11.0 | 0.00 | 1.00 | 1.00 | 11.0 | 0.00 | 1.00 | 1.00 | 11.0 | 0.00 | 1.00 | 1.00 | 11.0 | 0.00 | 1.00 | 1.00 |
| Education | -1.0 | 1.00 | 0.00 | 1.00 | -1.0 | 1.00 | 0.00 | 1.00 | -1.0 | 1.00 | 0.00 | 1.00 | -1.0 | 1.00 | 0.00 | 1.00 | -1.0 | 1.00 | 0.00 | 1.00 |
| *Schwerg et al.: ≥1* | 0.5 | 0.86 | 0.56 | 1.42 | 0.5 | 0.86 | 0.53 | 1.40 | 0.5 | 0.86 | 0.25 | 1.11 | 0.5 | 0.86 | 0.72 | 1.59^#^ | 0.5 | 0.86 | 0.68 | 1.54^#^ |
|  | 1.5 | 0.55 | 0.93 | 1.47^#^ | 1.5 | 0.55 | 0.93 | 1.48^#^ | 1.5 | 0.55 | 0.80 | 1.35^#^ | 1.5 | 0.55 | 1.00 | 1.55 | 1.5 | 0.55 | 0.97 | 1.51 |
|  | 2.5 | 0.09 | 0.98 | 1.07 | 2.5 | 0.09 | 1.00 | 1.09 | 2.5 | 0.09 | 0.90 | 0.99 | 2.5 | 0.09 | 1.00 | 1.09 | 2.5 | 0.09 | 1.00 | 1.09 |
|  | 4.0 | 0.00 | 1.00 | 1.00 | 4.0 | 0.00 | 1.00 | 1.00 | 4.0 | 0.00 | 1.00 | 1.00 | 4.0 | 0.00 | 1.00 | 1.00 | 4.0 | 0.00 | 1.00 | 1.00 |
| Criminal History | -1.0 | 1.00 | 0.00 | 1.00 | -1.0 | 1.00 | 0.00 | 1.00 | -1.0 | 1.00 | 0.00 | 1.00 | -1.0 | 1.00 | 0.00 | 1.00 | -1.0 | 1.00 | 0.00 | 1.00 |
| *Schwerg et al.: ≥1* | 0.5 | 0.77 | 0.65 | 1.43^#^ | 0.5 | 0.77 | 0.47 | 1.24^#^ | 0.5 | 0.77 | 0.20 | 0.97 | 0.5 | 0.77 | 0.83 | 1.61^#^ | 0.5 | 0.77 | 0.94 | 1.71^#^ |
|  | 1.5 | 0.27 | 0.87 | 1.14 | 1.5 | 0.27 | 0.87 | 1.14 | 1.5 | 0.27 | 0.55 | 0.82 | 1.5 | 0.27 | 1.00 | 1.27 | 1.5 | 0.27 | 1.00 | 1.27 |
|  | 2.5 | 0.00 | 0.95 | 0.95 | 3.0 | 0.00 | 1.00 | 1.00 | 2.5 | 0.00 | 0.80 | 0.80 | 3.0 | 0.00 | 1.00 | 1.00 | 3.0 | 0.00 | 1.00 | 1.00 |
|  | 4.0 | 0.00 | 1.00 | 1.00 |  |  |  |  | 4.0 | 0.00 | 1.00 | 1.00 |  |  |  |  |  |  |  |  |

| Table S2. (continued) | | | | | | | | | | | | | | | | | | | | |
| --- | --- | --- | --- | --- | --- | --- | --- | --- | --- | --- | --- | --- | --- | --- | --- | --- | --- | --- | --- | --- |
| Substance Use | -1.0 | 1.00 | 0.00 | 1.00 | -1.0 | 1.00 | 0.00 | 1.00 | -1.0 | 1.00 | 0.00 | 1.00 | -1.0 | 1.00 | 0.00 | 1.00 | -1.0 | 1.00 | 0.00 | 1.00 |
| *Schwerg et al.: ≥1* | 0.5 | 0.18 | 0.88 | 1.06^#^ | 0.5 | 0.18 | 0.87 | 1.05^#^ | 0.5 | 0.18 | 0.75 | 0.93 | 0.5 | 0.18 | 0.94 | 1.13^#^ | 0.5 | 0.18 | 0.94 | 1.12^#^ |
|  | 1.5 | 0.00 | 0.99 | 0.99 | 2.0 | 0.00 | 1.00 | 1.00 | 2.0 | 0.00 | 1.00 | 1.00 | 1.5 | 0.00 | 0.94 | 0.94 | 2.0 | 0.00 | 1.00 | 1.00 |
|  | 3.0 | 0.00 | 1.00 | 1.00 |  |  |  |  |  |  |  |  | 3.0 | 0.00 | 1.00 | 1.00 |  |  |  |  |
| Employment and Income | -1.0 | 1.00 | 0.00 | 1.00 | -1.0 | 1.00 | 0.00 | 1.00 | -1.0 | 1.00 | 0.00 | 1.00 | -1.0 | 1.00 | 0.00 | 1.00 | -1.0 | 1.00 | 0.00 | 1.00 |
| *Schwerg et al.: ≥1* | 0.5 | 0.68 | 0.48 | 1.16 | 0.5 | 0.68 | 0.40 | 1.08 | 0.5 | 0.68 | 0.15 | 0.83 | 0.5 | 0.68 | 0.44 | 1.13 | 0.5 | 0.68 | 0.74 | 1.42* |
|  | 1.5 | 0.18 | 0.99 | 1.17^#^ | 1.5 | 0.18 | 0.93 | 1.12^#^ | 1.5 | 0.18 | 1.00 | 1.18^#^ | 1.5 | 0.18 | 1.00 | 1.18^#^ | 1.5 | 0.18 | 1.00 | 1.18 |
|  | 3.0 | 0.00 | 1.00 | 1.00 | 3.0 | 0.00 | 1.00 | 1.00 | 3.0 | 0.00 | 1.00 | 1.00 | 3.0 | 0.00 | 1.00 | 1.00 | 3.0 | 0.00 | 1.00 | 1.00 |
| Living Situation | -1.0 | 1.00 | 0.00 | 1.00 | -1.0 | 1.00 | 0.00 | 1.00 | -1.0 | 1.00 | 0.00 | 1.00 | -1.0 | 1.00 | 0.00 | 1.00 | -1.0 | 1.00 | 0.00 | 1.00 |
| *Schwerg et al.: ≥1* | 0.5 | 0.55 | 0.81 | 1.35^#^ | 0.5 | 0.55 | 0.93 | 1.48^#^ | 0.5 | 0.55 | 0.95 | 1.50^#^ | 0.5 | 0.55 | 0.61 | 1.16 | 0.5 | 0.55 | 0.77 | 1.32^#^ |
|  | 1.5 | 0.41 | 0.89 | 1.30 | 1.5 | 0.41 | 0.93 | 1.34 | 1.5 | 0.41 | 0.95 | 1.36 | 1.5 | 0.41 | 0.83 | 1.24^#^ | 1.5 | 0.41 | 0.87 | 1.28 |
|  | 3.0 | 0.00 | 1.00 | 1.00 | 3.0 | 0.00 | 1.00 | 1.00 | 3.0 | 0.00 | 1.00 | 1.00 | 3.0 | 0.00 | 1.00 | 1.00 | 3.0 | 0.00 | 1.00 | 1.00 |
| Mental Health | -1.0 | 1.00 | 0.00 | 1.00 | -1.0 | 1.00 | 0.00 | 1.00 | -1.0 | 1.00 | 0.00 | 1.00 | -1.0 | 1.00 | 0.00 | 1.00 | -1.0 | 1.00 | 0.00 | 1.00 |
|  | 0.5 | 0.68 | 0.45 | 1.13 | 0.5 | 0.68 | 0.20 | 0.88 | 0.5 | 0.68 | 0.25 | 0.93 | 0.5 | 0.68 | 0.06 | 0.74 | 0.5 | 0.68 | 0.94 | 1.62 |
| *Schwerg et al.: ≥2* | 1.5 | 0.50 | 0.75 | 1.25^#^ | 1.5 | 0.50 | 0.67 | 1.17^#^ | 1.5 | 0.50 | 0.65 | 1.15^#^ | 1.5 | 0.50 | 0.50 | 1.00 | 1.5 | 0.50 | 1.00 | 1.50^#^ |
|  | 2.5 | 0.18 | 0.93 | 1.11 | 2.5 | 0.18 | 0.93 | 1.12 | 2.5 | 0.18 | 0.85 | 1.03 | 2.5 | 0.18 | 0.89 | 1.07^#^ | 2.5 | 0.18 | 1.00 | 1.18 |
|  | 4.0 | 0.00 | 1.00 | 1.00 | 4.0 | 0.00 | 1.00 | 1.00 | 4.0 | 0.00 | 1.00 | 1.00 | 4.0 | 0.00 | 1.00 | 1.00 | 4.0 | 0.00 | 1.00 | 1.00 |
| Sens Sensitivity, Spec Specificity. *Key Life History Domains, ^#^Youden cut-point | | | | | | | | | | | | | | | | | | | | |
